# Chemotherapy-Mediated Neuronal Aberration

**DOI:** 10.3390/ph16081165

**Published:** 2023-08-16

**Authors:** Pradip Kumar Jaiswara, Surendra Kumar Shukla

**Affiliations:** Department of Oncology Science, University of Oklahoma Health Science Centre, Oklahoma City, OK 73104, USA; pradip-jaiswara@ouhsc.edu

**Keywords:** chemotherapy, inflammation, cytokines, neuronal aberration, toxicity

## Abstract

Chemotherapy is a life-sustaining therapeutic option for cancer patients. Despite the advancement of several modern therapies, such as immunotherapy, gene therapy, etc., chemotherapy remains the first-line therapy for most cancer patients. Along with its anti-cancerous effect, chemotherapy exhibits several detrimental consequences that restrict its efficacy and long-term utilization. Moreover, it effectively hampers the quality of life of cancer patients. Cancer patients receiving chemotherapeutic drugs suffer from neurological dysfunction, referred to as chemobrain, that includes cognitive and memory dysfunction and deficits in learning, reasoning, and concentration ability. Chemotherapy exhibits neurotoxicity by damaging the DNA in neurons by interfering with the DNA repair system and antioxidant machinery. In addition, chemotherapy also provokes inflammation by inducing the release of various pro-inflammatory cytokines, including NF-kB, IL-1β, IL-6, and TNF-α. The chemotherapy-mediated inflammation contributes to chemobrain in cancer patients. These inflammatory cytokines modulate several growth signaling pathways and reactive oxygen species homeostasis leading to systemic inflammation in the body. This review is an effort to summarize the available information which discusses the role of chemotherapy-induced inflammation in chemobrain and how it impacts different aspects of therapeutic outcome and the overall quality of life of the patient. Further, this article also discusses the potential of herbal-based remedies to overcome chemotherapy-mediated neuronal toxicity as well as to improve the quality of life of cancer patients.

## 1. Introduction

**Figure 1:**
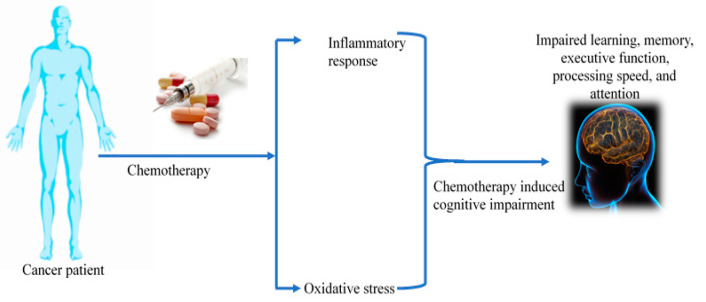
Effect of chemotherapy on patients’ brain.

Accumulating reports indicate that approximately 85% of cancer-treated patients agonize with long-term reduction in cognitive function that includes impaired memory, attention deficits, decreased multitasking, and executive function [1,2]. Chemotherapy is the fundamental therapeutic strategy for cancer treatment that is often associated with serious neurological and cogitative disorders referred to as chemotherapy-induced cognitive impairment (CICI)/chemo brain/chemo fog [3]. A wide spectrum of studies has examined the neurotoxic effect of various classes of chemotherapeutic agents, including alkylating agents, antimetabolites, microtubules, and tyrosine kinase inhibitors [4,5,6]. Chemo fog affects 75% of cancer-treated patients, and about 35% of patients exhibit post-treatment symptoms [7]. Chemotherapy-associated neurological accusations include neuropathy, encephalopathy, perceived mental slowness, vasculopathy, stroke, headache, and seizure [8,9,10], which are articulated as depression, anxiety, fatigue, pain, lack of concentration, and memory loss. Preclinical reports have shown that the administration of chemotherapeutic drugs such as cisplatin and doxorubicin to mice tumor models increases the cellular senescence markers [11]. Studies have shown that the use of platinum-based drugs for a wide range of solid tumors, including lung, bladder, and head and neck cancer, leads to cognitive flaws and structural irregularities in the brain [12,13,14]. The use of chemotherapeutic agents predominantly focuses on the induction of cancer cell death and also alters cytokine levels [15]. An elevation in inflammatory cytokines is correlated with cognitive dysfunction [5]. More interestingly, chronic inflammation is a major factor responsible for cognitive impairment in neurodegenerative ailments. Chemotherapeutic agents govern various cognitive functions by regulating pro-inflammatory cytokines in the frontal cortex, hippocampus, and corpus callosum [5]. A few animal studies observed the increased expression of various pro-inflammatory cytokines such as IL-6 and TNF-α in chemobrain mice. Elevated inflammatory response results in neurodegeneration and structural alteration in brain tissue. During chemotherapy treatment, pro-inflammatory cytokines (TNF-α, IL-6, IL-1, IL-1β) cross the blood-brain barrier via the peripheral nervous system [16]. Further, cancer patients treated with combinatorial chemotherapy of cyclophosphamide, methotrexate, and fluorouracil (CMF) experienced cognitive dysfunction not only after the treatment but also after 20 years [17]. The role of chemotherapeutic drug-mediated inflammation in the cognitive impairment of cancer patients is still under investigation. In this review, we discuss the effect of chemotherapeutic agents on physiological as well as molecular aberration of neuronal cells and their impact on brain function. An overview of chemotherapy-mediated brain abnormalities is provided in Figure 1.

## 2. Mechanism of Chemotherapy-Induced Cancer Cell Death and Inflammation

Chemotherapy is the most often used cancer treatment strategy [18]. The chemotherapeutic agent acts on the genomic content of tumor cells and exhibits cytotoxicity [19]. Cytotoxicity of chemotherapeutic agents is accompanied by interfering with various cellular processes, including cell division, DNA synthesis, and microtubule formation, which activates various cascades [18]. Chemotherapeutic agent activates apoptosis, a genetically programmed cell death mechanism, either via caspase-dependent and independent pathway or both together [20,21]. For example, the chemotherapeutic drug cisplatin and doxycycline induce both caspase-dependent and independent pathways [22,23], while paclitaxel activates only caspase-dependent apoptosis [24]. Fascinatingly, cisplatin and 5-fluorouracil also activate necroptosis, an immune cell-dependent cell death mechanism in tumor cells [25,26,27]. Moreover, chemotherapeutic agents also induced senescence by DNA damage. Briefly, chemotherapy-induced caspase-dependent apoptosis complements caspase-independent apoptosis, senescence, necroptosis, and autophagy [18]. It has been well established that chemotherapeutic drugs-induced cancer cell death occurs predominantly by elevating oxidative stress through enhancing ROS and RNS. A moderate elevation in oxidative stress regulates cell proliferation and differentiation, while excessive levels of oxidative stress damage biomolecules such as lipids, nucleic acids, and proteins, which contributes to cell death [28]. More than 50% of FDA-approved anticancer drugs increase oxidative stress through enhancing ROS/RNS that contribute to neuron cell death [29]. The elevated level of ROS disrupts the BBB by activating various pathways, including oxidative stress-mediated signaling [30]. Disruption of the BBB allows the entry of proinflammatory cytokines synthesized by chemotherapy-targeted tissue [31]. More interestingly, many studies reported that ROS/RNS-generating chemotherapeutic drugs dysregulate cytokines [15]. Further, cytokines enter the brain through receptor-mediated endocytosis or passive diffusion through the disrupted region of the BBB [29]. Moreover, toll like receptor 4 (TLR 4) is a possible candidate to maintain the BBB integrity after chemotherapy. The chemotherapy-induced oxidative stress activates the TLR4 pathways, which produces the proinflammatory cytokines TNF-α. As consequence, activation of intracellular NF-kB takes place [32]. Further, activated NF-kB triggers iNOS, which contributes more oxidative stress, finally leading to mitochondrial dysfunction and release of cytochrome c. Additionally, activated NF-kB also increases the proapoptotic protein (BAX and p53) and decreases antiapoptotic protein (BCL2). Consequently, activation of the apoptotic cascade results in neuron cell apoptosis in the brain [29]. Another molecular mechanism associated by CICI is inactivation of apolipoprotein A-1 (ApoA1). Basically, ApoA1 blocks the over-synthesis of TNF-α and IL-1β by activating JAK/STAT3 pathways [15,29]. However, oxidative stress-mediated oxidization of ApoA1 and disruption of JAK/STAT3 impaired its ability to inhibit TNF-α synthesis [15,29,33], leading to elevation in TNF-α, which further activates NF-kB. Therefore, excessive oxidative stress leads to neuronal cell death. Furthermore, TNF-α also activates the microglia cells by binding TNF-α receptors 1 and 2 of microglial cells and augments the inflammatory signal by local TNF-α production in the brain [34]. Additionally, activated microglia cells also elevate the ROS level by increasing the expression and activity of NADPH oxidase, primarily NOX 4 [34,35]. Hence, here we can conclude that a possible molecular mechanism behind CICI is chemotherapy-mediated inflammation and oxidative stress, which further elevate oxidative stress and finally lead to neuronal cell death.

## 3. Role of Commonly Used Chemotherapeutic Drugs in Neuronal Function

### 3.1. Doxorubicin

Doxorubicin is a member of the anthracycline drug family and exhibits a potent antitumor effect as a single and as an adjuvant therapeutic remedy [36]. Doxorubicin exhibits tumoricidal activity by intercalating DNA and inhibiting the activity of topoisomerase II [3,37]. Moreover, doxorubicin also induces the generation of reactive oxygen species that contribute to its tumoricidal activity [3]. Doxorubicin has a limited ability to cross the blood-brain barrier (BBB) despite its exhibiting significant neurotoxicity to the brain [3]. The permeability of doxorubicin to the BBB is restricted, while some studies suggest the potent antitumor activity of doxorubicin against brain tumors [3]. Several clinical studies revealed impaired cognitive assessments in cancer patients receiving doxorubicin [38,39,40]. In a study, it was observed that doxorubicin accumulated in the nuclei of neurons, leading to crosslinking of DNA, and ultimately induced DNA double-strand breaks (DSBs) [41]. Moreover, doxorubicin also hampers the DNA repair machinery by downregulating the DNA repair protein breast cancer type 1 susceptible (BRACA1) in primary cortical neurons, consequently inducing mitochondrial-mediated apoptosis in neuronal cells [41]. In addition, Wetzel et al. reported doxorubicin-mediated extrinsic or death receptor-mediated apoptosis of primary cortical neurons [42]. Further, reports also suggest increased ROS production and depolarization of mitochondrial membranes in doxorubicin-exposed neuronal cells [40,43]. Shokoohina et al. observed doxorubicin-induced neuronal degeneration by elevating the BAX/BCL2 ratio and mitochondrial outer membrane potential (MOMP) [43]. Ramalingayya et al. demonstrated that doxorubicin treatment was not only responsible for morphological anomalies in cell bodies, such as chromatin condensation and cell membrane destruction, but effectively suppressed neurite outgrowth in differentiated neuronal cell lines, which resulted in decreased neurite number and synapsin expression [40,41].

### 3.2. Effect of Doxorubicin-Mediated Oxidative Stress and Inflammation in Cognitive Impairment

In doxorubicin-induced chemobrain pathogenesis, inflammation and peripheral oxidative stress are well-recognized events [33,44]. Doxorubicin is a quinone-containing molecule, susceptible to one-electron reduction. NADPH cytochrome and P450 reductase catalyzed the doxorubicin in reduced semiquinone form [45]. Further, this semiquinone reacts with oxygen and produces superoxide radical (O^2−^), which acts as a source of ROS generation and leads to oxidative stress in the periphery [46,47]. Further, in vivo and clinical studies revealed that doxorubicin-mediated oxidative stress participates in the oxidation of biomolecules such as proteins, nucleic acids, and lipids. Moreover, doxorubicin also decreased the level of enzymatic and non-enzymatic antioxidant molecules [33,44,48]. Several studies suggest that reactive oxygen species effectively activate the nuclear factor NF-kB pathway [49,50,51]. The activated NF-kB pathway regulates the expression of various pro-inflammatory molecules, including tumor necrosis factor-α (TNF-α) in adaptive and innate immune cells [50]. Interestingly, it has been observed that intraperitoneal administration of doxorubicin elevated the plasma TNF-α level. More so, an increased TNF-α secretion was observed on incubation of mouse macrophage with serum collected from doxorubicin-administered mice [52,53]. Apolipoprotein A-1 (ApoA-1) is an anti-inflammatory molecule and negatively regulates TNF-α release from immune cells [54]. More interestingly, doxorubicin administration in mice declined the level of the anti-inflammatory molecule apolipoprotein A-1 (ApoA-1). Therefore, an increase of TNF-α in the circulation of doxorubicin-treated animals [40,52]. Thus, a decrease in ApoA-1 and an increase in ROS activate peripheral immune cells to release TNF-α. Circulating TNF-α bind to their receptors located on the endothelial cells of BBB and enter in the brain, where they activate the microglial and astrocytes cell to produce TNF-α in the brain that results in translocation of NF-kB in the nucleus [55,56,57]. Further, activated microglial and astrocytes not only produced TNF-α but also significantly enhanced ROS production through increasing the expression of NADPX oxidase [35,58]. NF-kB-mediated brain inflammation leads to the expression of inducible nitric oxide synthase (iNOS), which results in nitrosative and oxidative stress in the brain [51,59,60]. Few animal-based studies demonstrated the augmentation of nitric oxide and iNOS in the brain of doxorubicin-administered animals [53,57,61]. Holley et al. and Tangpong et al. observed nitration of mitochondrial antioxidant enzyme MnSOD that synergized O_2_^•−^ production [62,63]. Thus, available evidence states that inflammation and oxidative stress after the doxorubicin treatment might exacerbate the severity of doxorubicin-induced neurotoxicity.

### 3.3. Role of Cisplatin in Chemobrain

Cisplatin is a platinum-based anti-cancer agent that acts as a chelator, binds with adenine and guanine residue, and induces apoptosis in cancerous cells. Published studies demonstrated that head and neck cancer patients treated with cisplatin showed cognitive impairments [64]. Andrienne et al. found that within 30 min of a low dose of cisplatin (0.1 μM) leads to loss of synapses and dendritic spines. In contrast, higher cisplatin dose (1 μM) exposure causes rapid loss of synapses and dendritic disintegration [64]. The passage of cisplatin for BBB is limited; transportation of cisplatin across the BBB is mediated by copper transporter 1 (CTR1). Copper transporter 1 is expressed by endothelial cells of the brain and neurons. Few reports suggested that cancer patients treated with cisplatin shows a diminished brain glucose metabolism, abnormal brain network, and cognitive difficulty [14,65,66]. Chiang et al. observed that cisplatin-treated mice have decreased working and spatial memory as well as executive function [2]. Simo et al. observed structural abnormalities in the white matter of the brain after platinum-based chemotherapy [14]. Some in vivo studies have observed loss of arborization in myelin basic protein (MBP+) fibers and a drop in white matter complexity in the cingulum by increased white matter coherency in mice [67,68]. Oligodendrocytes are more vulnerable to cisplatin compared to various cancer cell lines. In vitro and in vivo studies revealed that cisplatin mainly induces defects in mitochondrial DNA (mtDNA). Manohar et al. demonstrated that cisplatin-induced the apoptosis in the hippocampus of the brain by increasing the expression of pro-apoptotic proteins (BIK, BOK, BID) and decreasing the expression of antiapoptotic proteins BCL2 [69]. In addition, it potentially diminishes oxygen consumption activity. Dysfunction of mitochondria after Cisplatin exposure leads to increased iNOS and NF-kB, diminished expression of antioxidant molecules, and declined neuronal progenitor [70,71]. Jangra et al. found oxidative products such as malondialdehyde (MDA) and protein carbonyl in the hippocampus of cisplatin-treated mice, indicating oxidative stress in the hippocampus of mice [71].

### 3.4. Molecular Mechanism of Cisplatin-Mediated Inflammation

In vivo studies indicated that injection of cisplatin (5 mg/kg) in rats for 7 weeks induced the activation of NF-kB and expression of downstream inflammatory cytokines, leading to inflammation in rats [70]. However, another animal-based study did not show any inflammatory response in the brain identified in terms of TNF-α, IL-6, and IL-1β expression [68,72]. Cisplatin-induced inflammatory event possibly is a time and dose-dependent effect. Chotourou et al. manifested that chronic treatment of cisplatin at high dose provoke inflammation in vivo [70].

### 3.5. Role of Paclitaxel in Chemobrain

Paclitaxel (PTX) is a microtubule stabilizing agent, which is often used in the first-line treatment for prevalent cancer types such as ovarian and breast cancer [73]. Paclitaxel displays tumoricidal activity by stabilizing the microtubule, causing cell cycle arrest and apoptosis [74]. In addition to therapeutic function, paclitaxel is responsible for peripheral neuropathy [75]. The paclitaxel permeability for BBB is limited, while positron emission tomography detected radiolabeled paclitaxel in brain tissue after intravenous administration [76]. This observation indicated that a small amount of paclitaxel crosses the BBB and induces apoptosis in neuron cells via stimulating endoplasmic reticulum stress [77]. The limbic system area, hippocampus, striatum, and cortex are major centers for cognitive processes (learning and spatial memory). Interestingly, the report also suggests that paclitaxel-mediated apoptosis of hippocampus cells causes impairment of neurological process [76].

### 3.6. Paclitaxel and Inflammation in Chemobrain

Paclitaxel administration impaired spatial learning and memory by increasing the TNF-α [76]. Paclitaxel augmented the TNF-α and IL-1β in hippocampus tissue, causing hippocampus neuronal apoptosis [76]. Recently, it has been observed that paclitaxel-induced cognitive impairment by decreasing the density of dendritic spines, BDNF, and PSD95. Paclitaxel increased necroptosis in hippocampal neurons by elevating the expression of receptor-interacting protein kinase 3 (RIP3). Animal treated with PTX shows an elevating level of inflammatory molecules (iNOS, TNF-α, IL-β), which leads to microglia polarization to M1 involved in cognitive impairment [78]. PTX triggers p38 MAPK/NF-κB signaling in peripheral monocytes and macrophages [79]. Further, NF-κB activates the expression of other pro-inflammatory cytokines such as TNF-α, IL-6, and IL-1β [80]. The circulating TNF-α from peripheral macrophages, and monocytes upsurge the penetrability of BBB. Consequently, paclitaxel and TNF-α can cross the BBB and act on the central nervous system (CNS) [76]. Further, TNF-α triggers an inflammatory response and NF-κB signaling in neurons, microglia, and astrocytes. Few reports suggested the apoptosis of local neuronal cells by NF-κB- TNF-α facilitated neuroinflammatory responses [81,82]. Zhao Li et al. found improvement in paclitaxel-induced spatial learning and memory impairment by thalidomide mediated TNF-α inhibition [76].

### 3.7. 5-Fluorouracil in Chemobrain

5-fluorouracil (5-FU) is a pyrimidine analog, a common anti-cancer drug of the antimetabolite class [83]. 5-FU is used to treat various cancers, including gastrointestinal, prostate, cervical, and vaginal cancer [84,85,86]. It induces programmed cell death in cancer cells by inhibiting the synthesis of DNA and RNA [84]. An in vivo study revealed that intraperitoneal administration of 5-FU impaired learning and memory [84]. 5-FU cross the blood-brain barrier by passive diffusion and inhibit the proliferation of hippocampus cell and inhibit hippocampus neurogenesis [87]. Treatment of breast cancer patients with 5-FU exhibits neurocognitive problems [88]. Mustafa et al. demonstrated that 5-FU exposure affects spatial working memory and disrupts the neurogenesis in the murine hippocampus [86]. Moreover, Thomas et al. found remarkable inhibition of dendritic branch point, dendritic length, and complexity in cornu ammonis (CA) and dentate gyrus (DG) after 5-FU treatment. Additionally, 5-FU declined the arborization in the dendritic area of CA and DG, which is associated with an impairment of hippocampus accompanying learning and memory [84].

### 3.8. Inflammatory Role of 5-FU in Chemobrain

Groves et al. demonstrated that administration of 5-FU in mice elevated the expression of both pro-inflammatory and anti-inflammatory cytokines. Administration of 5-FU increased the expression of pro-inflammatory cytokines such as IL-17, IL-1β, and GM-CSF [84]. IL-1β plays a role in hippocampus-dependent memory, while IL-17 plays a pivotal role in the pathogenesis of inflammation-associated diseases of the central nervous system (CNS), such as stroke and sclerosis [89]. Few clinical studies also shown that chemotherapy-induced IL-17, IL-1β, and GM-CSF expression in cancer patients are more prominent who faced cognitive impairment [90,91]. The constant activation of microglial cells and secretion of IL-1β could cause damage to glial and neuronal cells [90]. More interestingly, 5-FU administration also augments the expression of anti-inflammatory cytokines IL-2, IL-3, IL-4, and IL-5, which play a role in the proliferation, survival, and protection of neuronal cells [84]. The anti-inflammatory activity of IL-4 might be because of antagonizing the inflammatory response of IL-1β [84,92]. Interestingly, it has been observed that 5-FU administration arouses the expression of one chemokine RANTES (regulated upon activation of normal T cell expressed and secreted) which is immunologically designated as CCL5 [84]. CCL5 activates the recruitment of immune cells in the CNS [93] and increases the expression of genes involved in synaptogenesis, neurite outgrowth, and neuronal survival [94].

## 4. Combinatorial Chemotherapy in Chemobrain

**Figure 2:**
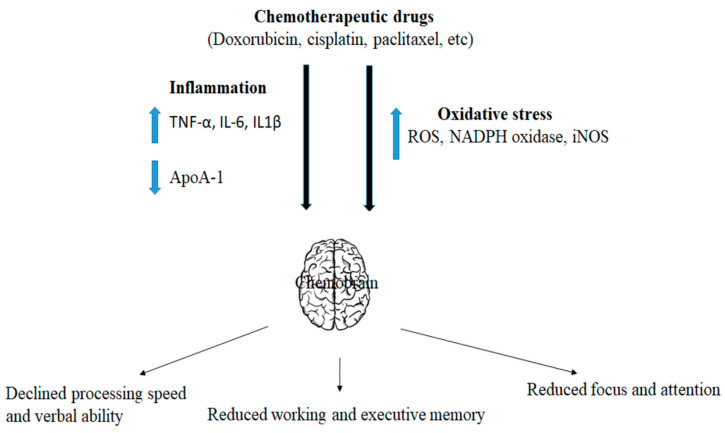
Molecular mediators of chemotherapy-mediated neuronal abnormalities.

**Figure 3:**
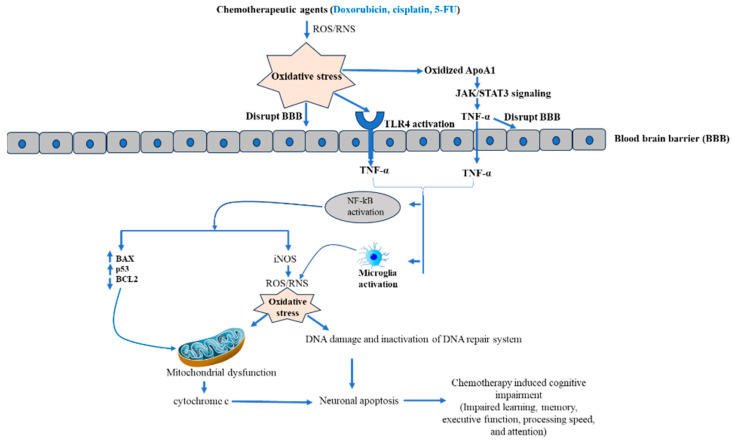
Molecular mechanism of chemotherapy-mediated neuro-toxicity.

A combinatorial therapeutic approach is a strategy of combining two or more drugs for a more positive outcome. Combinatorial therapeutic approaches have emerged as superior to monotherapy against many cancers [95]. However, many reports suggest the impact of combinatorial chemotherapy on chemobrain. Anderson et al. observed that a combination of cyclophosphamide, methotrexate, and 5-fluorouracil (CMF) in mice exhibits long-term memory impairment [96]. Further, another in vivo report revealed that intraperitoneal injection of 5-fluorouracil and methotrexate to rats induced cognitive impairment and suppressed neurogenesis [97]. Shi et al. detected that intraperitoneal administration of docetaxel, adriamycin, and cyclophosphamide exhibits impairment of memory and hippocampal neuronal activity [98]. The combination of another two drugs, adriamycin, and cyclophosphamide (AC), in C57BL/6 mice display memory impairment and decreased the total dendritic length, spines density, and hippocampal neuron maturation [99]. A brief overview of molecular mediators of chemotherapy-induced neuronal toxicity has been presented in Figure 2 and their impact on signaling pathways has been presented in Figure 3.

## 5. Possible Therapeutic Intervention for Chemotherapy-Mediated Chemobrain

### 5.1. Phytochemical as Chemobrain Therapeutic Intervention

The use of phytochemicals is a well-established therapeutic approach against various ailments having minimal side-effect, better biocompatibility, easy availability, and cost-effectiveness [100,101]. Phytochemicals have potent tumoricidal activity against most cancers [102]. Moreover, previous reports also claim the neuroprotective properties of phytochemicals against chemotherapy-induced neurotoxicity [103]. Mohamed et al. found that Epicatechin, a polyphenolic molecule from green tea, supplemented orally at dosage of 10 mg/kg/day for 2 weeks prior to doxorubicin injection and then for another 2 weeks with doxorubicin. They found significant neuroprotective activity of epicatechin against doxorubicin-induced neuronal toxicity. Doxorubicin treatment increases the peripheral TNF-α, which crosses the BBB and induces various inflammatory pathways as well as glial cell activation. Accordingly, more TNF-α production that leads to mitochondrial dysfunction and finally neuron death [61]. Indeed, long term utilization of doxorubicin can cause neurodegenerative disorder due to its continued activation of microglia, which enhances the synthesis of neurotoxic proinflammatory mediators, direct to neuronal cell death. Epicatechin effectively declined the level of TNF-α, NF-kB, iNOS, and lipid peroxidation along with augmentation of antioxidant enzymes in brain tissue of doxorubicin-administered mice [61]. Similarly, treatment of Xanthone, a biologically active compound of tropical fruit Mangosteen, rescue the mice from doxorubicin-mediated neuronal toxicity by inhibiting the doxorubicin-induced elevated expression of pro-apoptotic proteins, level of circulating TNF-α, and oxidative stress in doxorubicin treated mice [53]. This bioactive compound acts as free radicals scavenger, which is a crucial feature to encounter the doxorubicin-induced chemobrain.

Naringin, a bioactive flavonoid of citrus juice from grapefruit, exhibits therapeutic activity such as anti-cancer and antioxidant [104,105]. Chtourou et al. orally administered the naringin at dosage of 25 mg, 50 mg, and 100 mg/kg/week with cisplatin (5 mg/kg/week) for five consecutive weeks and detected improved gross motor and neurobehavior impaired by cisplatin [70]. Cisplatin treatment remarkably elevated the acetylcholinesterase and inducible nitric oxide synthase (iNOS) in hippocampus while declined the protein carbonyls (PCO), reactive oxygen species (ROS), nitrite formation (NO), and malondialdehyde (MDA) that contribute to cisplatin associated chemobrain. However, naringin treatment prevented all the biochemical and molecular alteration in cisplatin treated mice [70]. Further, the possible therapeutic potential of naringin against doxorubicin induced chemobrain can be achieve, as one study revealed the potent role of naringin in diminishing the doxorubicin induced oxidative and nitrosative stress in mice [106]. Thus, the pleiotropic role of naringin directs it as an effective therapeutic candidate for cognitive dysfunction in cancer patients.

Recently, John et al. demonstrated the neuroprotective effect of lumina mango, Mangifera indica fruit pulp, Curcuma longa rhizome, Centella asiatica, and nutrient and vitamins [107]. Various studies suggested the neuroprotective, anti-inflammatory, and antioxidant properties of mangiferin [108,109]. Mangiferin is a possible neuroprotective candidate against chemotherapy-induced neurotoxicity because of its ability to cross the blood-brain barrier [107]. In vivo studies revealed that combinatorial treatment of cyclophosphamide, Methotrexate, and 5-fluorouracil (CMF) reduced the burden of mammary cancer in mice and led to cognitive dysfunction. Daily oral administration of mulmina (40 mL/kg and 80 mL/kg) started one week before chemotherapy and continued till the end of chemotherapy cycle showed a remarkable reduction in tumor volume which revealed that mulmina did not impede the anti-cancer activity of CMF therapy [107]. Animals treated with 40 ml/kg mulmina display more learning behavior than animals treated with CMF. Pre-treatment of MN-40 & MN-80 to tumor-bearing animals subjected to chemotherapy displayed less oxidative damage than chemotherapy-treated animals. Moreover, mulmina improved the antioxidant system disrupted by CMF treatment. As we discussed, pro-inflammatory cytokines (IL-1β, IL-6, and TNF-α) play a role in neuroinflammation and cognitive impairment. It has been seen that CMF treatment enhances the IL-1β, IL-6, and TNF-α in the tumor-bearing animal. However, the augmented level of IL-1β, IL-6, and TNF-α was reversed after mulmina treatment [107]. In the quest of potential phytochemical for treatment of chemobrain, donepezil or rivastigmine, an acetylcholinesterase inhibitor, improves cognitive function [110]. In breast cancer survivors, after chemotherapy, a 5–10 mg/day of donepezil for 24 weeks (5 mg/day for six weeks and if tolerated increased the dosage to 10 mg/day for next 18 weeks) better the two parameter of memory- the Hopkin verbal learning test-revised (HVLT-R) and HVLT-R discrimination. However, there were no noticeable effect on other cognitive variables or in subjective cognitive function or quality of life [111]. Further, Shaw et al. operated a phase II open level study in irradiated brain tumor survivor treated with 5 mg/kg of donepezil for 6 weeks followed by 10 mg/kg for 18 weeks found improvement in cognitive attention, verbal and figural memory, mood, fatigue, and quality of life [112]. Donepezil is an FDA approved drug for Alzheimer disease. Moreover, many clinical and preclinical investigations established donepezil for CICI treatment [111,113]. Interestingly, donepezil has been identified for its recue ability for inflammation associated cognitive dysfunction. In vivo investigation indicated that donepezil inhibits CMF associated cognitive deficiency in mammary carcinoma model by reverting the increased level of proinflammatory cytokines (IL-6 and IL-1β) in CMF treated animal tumor model [107].

Curcumin, a bioactive molecule extracted from *Curcuma longa,* has tremendous medicinal value against a wide range of human ailments, including obesity, diabetes, and neurological disorders [114,115]. Clinical studies suggested that curcumin recovers cognitive deficits and is established as an efficacious, well-tolerated, and safe phytochemical [116,117]. Few studies demonstrated that curcumin was able to reverse the cognitive dysfunction induced by chronic mild stress and activate the proliferation of astrocyte cells of the striatum and hippocampus. Cisplatin-induced brain toxicity in rats by increasing the mitochondrial lipid peroxidation and protein carbonyl. However, treatment with curcumin (200 mg/kg) before 24 h cisplatin treatment (6 mg/kg) improved cisplatin-induced brain toxicity by rehabilitating mitochondrial lipid peroxidation and protein carbonyl [118]. Moreover, several reports documented that curcumin could reduce chemotherapy-induced brain toxicity by suppressing inflammatory and anti-inflammatory cytokines such as NF-κB, STAT3, COX, LO, and Xanthine oxidase [119]. Hence, curcumin’s inflammation regulatory ability may play an important therapeutic role in inflammation-induced chemobrain. Although, curcumin has well established safety proof, few adverse side effects have been detected. In a dose response study, seven subject after 72 h of receiving curcumin (500–1200 mg) suffered with headache, diarrhea, rash, and yellow stool [120,121]

Resveratrol, a non-flavonoid polyphenol naturally present in various species of plants, including grapes, peanuts, berries, and red wine [122,123,124]. Resveratrol exhibits anticancer activity against a wide variety of cancers such as prostate, skin, liver, ovarian, and lung cancers [125]. Moreover, studies also revealed the antioxidant, anti-inflammatory [125], immunomodulatory and neuroprotective role of resveratrol [126]. Shi et al. found that resveratrol protects combinatorial chemotherapy induced cognitive dysfunction. In vivo study revealed that oral administration of resveratrol (100 mg/kg/day) for three weeks, beginning one week prior the DAC treatment ameliorated the DAC (docetaxel, adriamycin, cyclophosphamide) induced cognitive impairment in mice. In addition, resveratrol treatment noticeably lowered the pro-inflammatory cytokines (TNF-α and IL-6) in DAC treated mice [126]. Recently, another in vivo study found that resveratrol treatment improved the cognitive function in mice impaired by paclitaxel treatment. Resveratrol treatment declined the apoptosis and oxidative stress by activating the SIRT1 and PGC-1α pathways that causes betterment of paclitaxel mediated cognitive dysfunction. Besides, resveratrol treatment also significantly elevated the level of anti-inflammatory cytokines (IL-4 and IL10) [127]. Since resveratrol is a plant bioactive molecule, it exhibits adverse effects also. Its low dosage exhibits advantageous effect while higher dosage shows toxicity. The lower concentration of resveratrol acts as antioxidant while on higher concentration behave as pro-oxidant [128]. Resveratrol does not exhibit side effects at short-term doses (1 gm). However, dose of 2.5 gm or more per day has side effects such as diarrhea, nausea, vomiting, and liver dysfunction in non-alcoholic fatty acid disease patient [129,130]. Interestingly, long term clinical trials have not detected any major side effects. Indeed, resveratrol at dose 5 g/day has been identified safe and well tolerated, either as a single dose or as fraction of multiple dose [130,131].

Another plant derived bioactive molecule to rescue the chemotherapy induced neuronal aberration is catechins, chemically designed as flavan-3-ol, derived from tea leaves. Catechins have several pharmacological values, including antioxidant, anti-inflammatory, and antitumor properties [132]. The neuroprotective role of catechins was evaluated at a dose of 100 mg/kg against the doxorubicin (DOX) induced memory deficit in animal model and found a protective efficacy for DOX associated memory deficit. Catechins treatment showed a remarkable decrease in oxidative stress and neuroinflammation in cerebral cortex and hippocampus in DOX treated animal [132]. Catechin declined the oxidative stress by restoring the antioxidant defense molecules, including SOD catalase and GSH.

### 5.2. Anti-Inflammatory Drug in Chemobrain Therapy

A series of studies examined the effect of anti-inflammatory drugs (naproxen, aspirin, and ibuprofen) on the treatment of Alzheimer’s disease (AD). Drugs such as cladribine, rituximab, and Copaxone restrict the migration of immune cells across the blood-brain barrier, able to reduce cognitive impairment [133]. PLX5622 is an inhibitor of the colony-stimulating factor 1 receptor, inhibits methotrexate-induced memory impairment. Moreover, PLX5622 inhibits inflammation and improves cognitive deficits in the Alzheimer’s mouse model [134].

PAN 811, chemically designed as 3-aminopyridine-2-carboxaldehyde thiosemicarbazone (3-AP) also referred as triapine, acts as inhibitor for ribonucleotide reductase [135]. PAN-811 is verified in phase 1 and 2 clinical trials for cancer treatment [97,136,137]. It has free radicals scavenging activity and inhibits H_2_O_2_ induced neuronal toxicity. Furthermore, 3-AP is well known for inhibiting the cell death induced by neurotoxic agents such as veratridine, glutamate, and staurosporine. In vitro study illustrated that 0.5 µm of 3-AP counteracts ischemic neurotoxicity [135]. Further, another in vitro study evidence that PAN 811 prevents the methotrexate (MTX) and 5-flurouracil (5-FU) mediated neurotoxicity by lowering the oxidative stress. MTX and 5-FU treatment impaired the cognitive function such as spatial memory, non-matching-to-sample rule-learning, and discrimination learning in mice [97]. The experimental evidence suggested that PAN 811 treatment improved cognitive dysfunction in animal treated with MTX/5-FU. Thus, PAN 811 prevents cognitive deficits resulting with MTX/5-FU treatment and sustain neurogenesis in dentate gyrus. An overview of different potential therapeutic interventions for chemobrain has been provided in Table 1.

## 6. Conclusions and Future Prospective

The possible molecular events underlying the chemobrain are chemotherapy-induced oxidative damage, mitochondrial dysfunction, and inflammation. All these processes are associated with chemobrain pathogenesis. Thus, chemobrain compromises the quality of life of cancer patients and limits the application of chemotherapy. Chemotherapeutic drugs are not easily cross the blood-brain barrier. However, the drug-induced cytokines such as IL-1β, IL-6, and TNF-α cross the BBB and exhibit neurotoxicity as well as chemobrain. Thus, treatments with anti-inflammatory and antioxidative agents improve parameters remarkably. Based on accumulating evidence, possible potential agents to treat the chemobrain have been identified, including epicatechin, xanthone, alumina, curcumin, and anti-inflammatory drug (PLX5622). In addition to these molecules, which we discussed in this review, there are some more possible potential agents to cure the chemobrain, including Metformin, Lithium, Astaxanthin, and Fluoxetine. Although, extensive animal-based studies are required to explore the detailed mechanism associated with chemobrain. Over and above that, unambiguous clinical trials are needed to recognize the drug targets and their therapeutic efficacy.

## Figures and Tables

**Table 1:** Potential therapeutic interventions for chemotherapy-induced neuro toxicity.

Drug for Chemobrain Intervention	Role in Chemobrain	References
**Epicatechin**	Decline the TNF-α, NF-Kb, iNOS level Increased the level of antioxidant ezymes	[61]
**Xanthone**	Inhibits apoptotic protein, oxidative stress and TNF-α	[53]
**Naringin**	Inhibit oxidative stress by elevating antioxidant enzymes	[70]
**Mangiferin**	Decreases oxidative stress Suppress IL-1β, IL-6, and TNF-α level	[108]
**Curcumin**	Suppress NF-κB, STAT3, COX, LO	[119]
**Resveratrol**	Decline proinflammatory cytokines TNF-α and IL-6	[126]
**Catechin**	Restore antioxidant defence molecules (SOD, catalase, and GSH)	[132]
**Donepezil**	Decrease proinflammatory cytokines (IL-6 and IL-1β)	[107]

## Data Availability

Not applicable.
